# Nicotine aggravates pancreatic fibrosis in mice with chronic pancreatitis via mitochondrial calcium uniporter

**DOI:** 10.18332/tid/186587

**Published:** 2024-04-30

**Authors:** Xue Wei, Yue Yuan, Miaomiao Li, Zhiren Li, Xinye Wang, Haoxuan Cheng, Xinjuan Liu, Jianyu Hao, Tong Jin

**Affiliations:** 1 Department of Gastroenterology, Beijing Chaoyang Hospital, Capital Medical University, Beijing, China

**Keywords:** nicotine, pancreatic stellate cells, chronic pancreatitis, mitochondrial calcium homeostasis, oxidative stress

## Abstract

**INTRODUCTION:**

This study aimed to investigate the effects of nicotine on the activation of pancreatic stellate cells (PSCs) and pancreatic fibrosis in chronic pancreatitis (CP), along with its underlying molecular mechanisms.

**METHODS:**

This was an *in vivo* and *in vitro* study. *In vitro*, PSCs were cultured to study the effects of nicotine on their activation and oxidative stress. Transcriptome sequencing was performed to identify potential signaling pathways involved in nicotine action. And the impact of nicotine on mitochondrial Ca^2+^ levels and Ca^2+^ transport-related proteins in PSCs was analyzed. The changes in nicotine effects were observed after the knockdown of the mitochondrial calcium uniporter (MCU) in PSCs. *In vivo* experiments were conducted using a mouse model of CP to assess the effects of nicotine on pancreatic fibrosis and oxidative stress in mice. The alterations in nicotine effects were observed after treatment with the MCU inhibitor Ru360.

**RESULTS:**

*In vitro* experiments demonstrated that nicotine promoted PSCs activation, characterized by increased cell proliferation, elevated α-SMA and collagen expression. Nicotine also increased the production of reactive oxygen species (ROS) and cellular malondialdehyde (MDA), exacerbating oxidative stress damage. Transcriptome sequencing revealed that nicotine may exert its effects through the calcium signaling pathway, and it was verified that nicotine elevated mitochondrial Ca2+ levels and upregulated MCU expression. Knockdown of MCU reversed the effects of nicotine on mitochondrial calcium homeostasis, improved mitochondrial oxidative stress damage and structural dysfunction, thereby alleviating the activation of PSCs. *In vivo* validation experiments showed that nicotine significantly aggravated pancreatic fibrosis in CP mice, promoted PSCs activation, exacerbated pancreatic tissue oxidative stress, and increased MCU expression. However, treatment with Ru360 significantly mitigated these effects.

**CONCLUSIONS:**

This study confirms that nicotine upregulates the expression of MCU, leading to mitochondrial calcium overload and exacerbating oxidative stress in PSCs, and ultimately promoting PSCs activation and exacerbating pancreatic fibrosis in CP.

## INTRODUCTION

Chronic pancreatitis (CP) is a fibrotic disorder characterized by the progressive fibrosis of pancreatic tissue resulting from recurrent episodes of inflammation, impacting both the quality of life and lifespan of patients^1^. The incidence of CP has been steadily increasing, posing a significant economic and psychological burden on patients and their families due to the lack of effective treatments^2,3^. Various factors contribute to the development of CP, with smoking being a commonly encountered risk factor^4^. Nicotine, a component of tobacco, is believed to play a crucial role in exacerbating CP.

Pancreatic stellate cells (PSCs) play a pivotal role in the development of pancreatic fibrosis in CP, and their activation is considered an initial step in fibrotic progression^5^. In our previous study, we demonstrated that nicotine exacerbates pancreatic fibrosis in rats with CP and activates PSCs *in vitro*^6^. Furthermore, we observed that nicotine increases the levels of reactive oxygen species (ROS) in human pancreatic stellate cells (hPSCs) mitochondria, leading to upregulation of dynamin-related protein 1 (DRP1)^7^.

Mitochondria, as vital organelles, play a pivotal role in cellular function^8,9^. They regulate cell biology by maintaining the balance of ROS and calcium homeostasis^10-12^. Numerous studies have indicated a strong correlation between mitochondrial dysfunction and the development of various diseases^13-15^. Therefore, investigating mitochondrial function may provide new insights for the treatment of CP. Mitochondrial calcium homeostasis is intricately linked to the biological properties of cells^16^. Dysregulation of mitochondrial calcium homeostasis has been implicated in various diseases, including neurological disorders, diabetes, cancer, and other pathological conditions^17,18^.

Nevertheless, the impact of alterations in mitochondrial calcium homeostasis in CP remains unclear. Therefore, the aim of this study was to investigate the influence of alterations in calcium homeostasis on nicotine-aggravated pancreatic fibrosis in CP.

## METHODS

### Mice

This is an *in vivo* and *in vitro* study performed on mice. C57BL male mice, aged 6–8 weeks and weighing 20–25 g, were selected for *in vitro* and *in vivo* experiments. All animal experiments were conducted in strict compliance with the ARRIVE guidelines^19^. The National Research Council’s Guide for the Care and Use of Laboratory Animals was also followed to guarantee the appropriate care and ethical treatment of the animals involved.

### Cell culture and transfection

Mouse pancreatic stellate cells (mPSCs) were isolated and cultured in complete DMEM/F12 medium (Gibco). The experiments in this study utilized mPSCs at passages 1–2, which were then stimulated with nicotine (Sigma Aldrich). In addition, mPSCs were transfected with small interfering RNA (siRNA) obtained from Syngentech (China) using Lipofectamine RNAiMAX (Invitrogen).

### Cell proliferation

Cell proliferation was assessed using the Cell Counting Kit-8 (CCK-8, Lablead, China). A total of 2.0×10^3^ mPSCs were seeded in each well of a 96-well plate. The absorbance at 450 nm was measured to quantify cell proliferation.

### Quantitative real-time polymerase chain reaction (qRT-PCR)

Total RNA was extracted from mPSCs and pancreatic tissues using TRIzol reagent (Invitrogen). Reverse transcription was performed using the PrimeScript RT Master Mix (Takara). qRT-PCR was conducted on an ABI 7500 system (Applied Biosystems) using the SYBR Green Master Mix and primers listed in Supplementary file Table 1. The relative mRNA levels were determined using the Livak (2-ΔΔCt) method.

### Assessment of mitochondrial reactive oxygen species (ROS)

A total of 2.0×10^3^ mPSCs were seeded in each well of a 96-well plate. Cells were incubated in 500 nM MitoSOX Red (Invitrogen) for 30 min and Hoechst 33342 (Beyotime Biotechnology, China) for 10 min, protected from light. Fluorescence intensity of Hoechst (ex/em: 350/461 nm) and MitoSOX (ex/em: 396/610 nm) were detected, respectively. The intensity of MitoSOX was normalized to that of Hoechst.

### Measurement of malondialdehyde (MDA)

MDA levels in mPSCs and serum were quantified using the MDA assay kit (NJJCBIO, China). After adding the relevant reagents and incubating at 95^o^C for 40 min, the absorbance at 530 nm was determined.

### RNA sequencing (RNA-seq)

RNA-seq was performed by Novogene Co., Ltd. (Beijing, China). Briefly, total RNA was extracted from mPSCs using TRIzol reagent (Invitrogen). Qualified libraries were sequenced on the Illumina NovaSeq 6000 platform. Clean reads were aligned to the reference genome using Hisat2 (v2.0.5). The gene expression levels were estimated by calculating the expected number of Fragments Per Kilobase of transcript sequence per Millions base pairs sequenced (FPKM) for each gene. We utilize the Gene Set Enrichment Analysis (GSEA) analysis tool available at http://www.broadinstitute.org/gsea/index.jsp for conducting GSEA.

### Assessment of mitochondrial Ca2+ levels

Cells were treated with 4 μM Rhod-2 AM (Invitrogen) for 20 min and Hoechst 33342 (Beyotime) for 10 min, both in a light-protected environment. Subsequently, fluorescence microscopy was employed to visualize the signals. The intensity of Rhod-2 was normalized to that of Hoechst.

### Western blotting analysis

Protein extraction from mPSCs and pancreatic tissues was lysed using RIPA buffer (Solarbio) supplemented with protease and phosphatase inhibitors. The protein concentration was determined using the BCA Assay Kit (Beyotime). Protein samples underwent SDS-PAGE electrophoresis and were transferred onto PVDF membranes. Subsequently, the membranes were incubated overnight at 4^o^C with the primary antibody, followed by a 1-h incubation with the secondary antibody. Details of the antibodies used are provided in Supplementary file Table S2.

### Assessment of mitochondrial membrane potential (MMP)

MMP was assessed using the Mito Probe JC-1 assay kit (Invitrogen). Cells were incubated with 2 μM JC-1 for 15–30 min. The red and green signals of JC-1 were visualized using a fluorescence microscope. The ratio of red/green fluorescence intensity was calculated as a measure of MMP.

### CP model and treatment

The CP model was established by repeated intraperitoneal injections of cerulein, 50 μg/kg, six times per day with a 1-hour interval, three days a week for four weeks^20^. Nicotine was administered via intragastric administration at a dose of 6 mg/kg/day, four times a day for four weeks^6,21^. Ru360 was administered by intraperitoneal injection, 240 μg/kg, one hour before nicotine, once a day for four weeks^22^.

### Histology

Pancreas tissues were fixed in 4% paraformaldehyde. The degree of pancreatic fibrosis was assessed using hematoxylin and eosin (H&E), Masson trichrome, and Sirius red staining performed by Servicebio technology.

### Immunohistochemical staining

Immunohistochemical staining of α-SMA in pancreatic tissue was performed to assess the activation of mPSCs. The slides were first incubated with α-SMA antibody (Abcam, #ab5694) overnight at 4°C. Subsequently, the slides were incubated with horseradish peroxidase-conjugated secondary antibodies (ZSGB-BIO) for 20 min.

### Statistical analysis

The results are presented as mean ± standard deviation (SD). Statistical analysis was performed using the unpaired t-test or one-way ANOVA with SPSS software (version 24.0, Chicago, USA). A p<0.05 was considered statistically significant.

## RESULTS

### Nicotine promotes activation and induces mitochondrial oxidative stress in mPSCs

**Figure 1:**
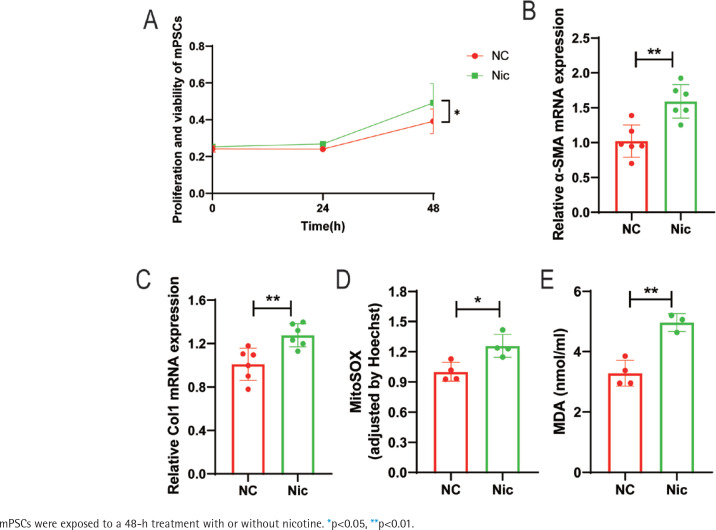
Nicotine promotes activation and induces mitochondrial oxidative stress in mouse pancreatic stellate cells (mPSCs): A) Relative proliferation and viability of mPSCs determined by CCK-8 assays; B, C) mRNA levels of α-SMA and Col1 in mPSCs; D) Mitochondrial reactive oxygen species (ROS) levels in mPSCs; E) Intracellular malondialdehyde (MDA) levels in mPSCs

In our previous study, we discovered that nicotine has the ability to enhance the activation of hPSCs and induce mitochondrial oxidative stress. The most significant impact was observed when cells were treated with 1μM nicotine for 48 hours^7^. In this study, we extracted and cultured mPSCs *in vitro* to simulate the condition of PSCs in CP. We found that nicotine treatment (1 μM, 48 h) significantly promoted the activation of mPSCs, as evidenced by a notable increase in cell proliferation (p<0.05) (Figure 1A) and an upregulation in the mRNA expression level of α-SMA, a marker of PSCs activation (p<0.01) (Figure 1B). Additionally, nicotine also stimulated fibroblast activity in mPSCs and elevated the mRNA expression level of Type I collagen (Col I), the primary constituent of the extracellular matrix (ECM), suggesting that nicotine may disrupt ECM metabolism in mPSCs (p<0.01) (Figure 1C).

Furthermore, nicotine aggravated mitochondrial oxidative stress in mPSCs. The levels of mitochondrial ROS in the nicotine group were significantly elevated compared to the negative control group (p<0.05) (Figure 1D). Moreover, the level of malondialdehyde (MDA), a product of lipid peroxidation, was significantly increased (p<0.01) (Figure 1E). These findings indicate that nicotine may induce damage to mPSCs through oxidative stress.

### The potential involvement of the calcium signaling pathway in the promotion of mPSCs activation by nicotine

**Figure 2:**
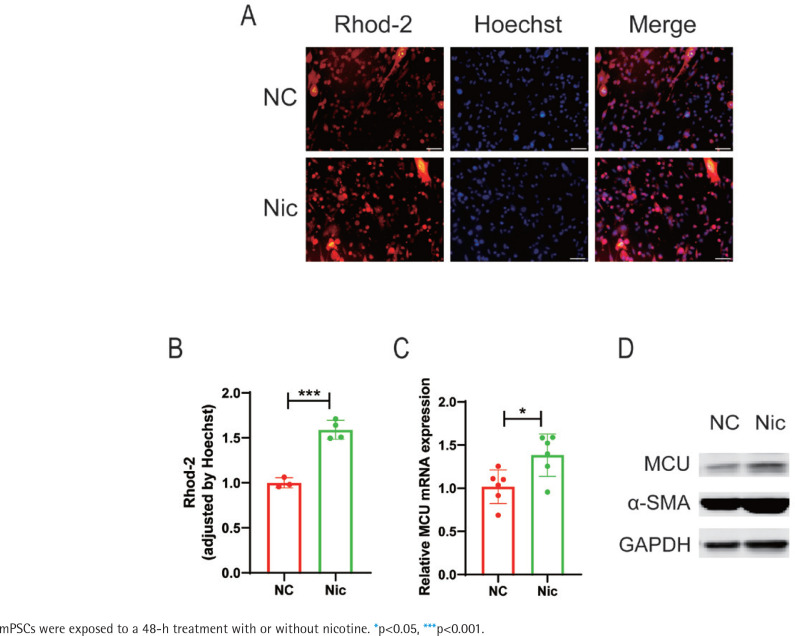
The potential involvement of the calcium signaling pathway in the promotion of mPSCs activation by nicotine: A) Fluorescent microscopy images showing Rhod-2 staining of mitochondrial Ca^2+^ and Hoechst staining of the cell nucleus in mPSCs (scale bars=100 μm); B) Measurement of mitochondrial Ca^2+^ levels by normalizing the fluorescence intensity of Rhod-2 to that of Hoechst in mPSCs; C) mRNA levels of mitochondrial calcium uniporter (MCU) in mPSCs; D) Protein expression levels of α-SMA and MCU

To investigate the underlying mechanism by which nicotine promotes mPSCs activation, transcriptome sequencing was conducted comparing the nicotine-treated group with the negative control group of mPSCs. Principal component analysis (PCA) revealed a significant distinction between the two groups (Supplementary file Figure S1A). GSEA indicated an upregulation of the calcium signaling pathway in the nicotine treatment group compared to the control group, suggesting that nicotine may exert its effects through this pathway (NES=1.272, p=0.046) (Supplementary file Figure S1B). Consequently, we examined mitochondrial Ca^2+^ levels in nicotine-treated mPSCs using Rhod-2 probes under fluorescence microscopy (Figure 2A). Notably, the mitochondrial Ca^2+^ level in mPSCs exhibited a significant increase upon nicotine stimulation (p<0.001) (Figure 2B), implying a potential link between nicotine-induced biological changes in mPSCs and alterations in mitochondrial calcium homeostasis.

Considering the association of nicotine’s effects on mPSCs with mitochondrial Ca^2+^ levels, we further investigated the impact of nicotine on proteins involved in mitochondrial calcium transport. Among these proteins, the mitochondrial calcium uniporter (MCU) plays a crucial role in facilitating mitochondrial calcium influx. Interestingly, our findings demonstrated a substantial elevation in MCU mRNA levels following nicotine stimulation (p<0.05) (Figure 2C), accompanied by an increasing trend in MCU protein expression (Figure 2D). These results suggest that nicotine modulates mitochondrial calcium homeostasis, potentially mediated by upregulating MCU, thus contributing to mPSCs activation.

### Nicotine aggravates mPSCs activation through the dysregulation of mitochondrial calcium homeostasis and oxidative stress mediated by MCU

**Figure 3:**
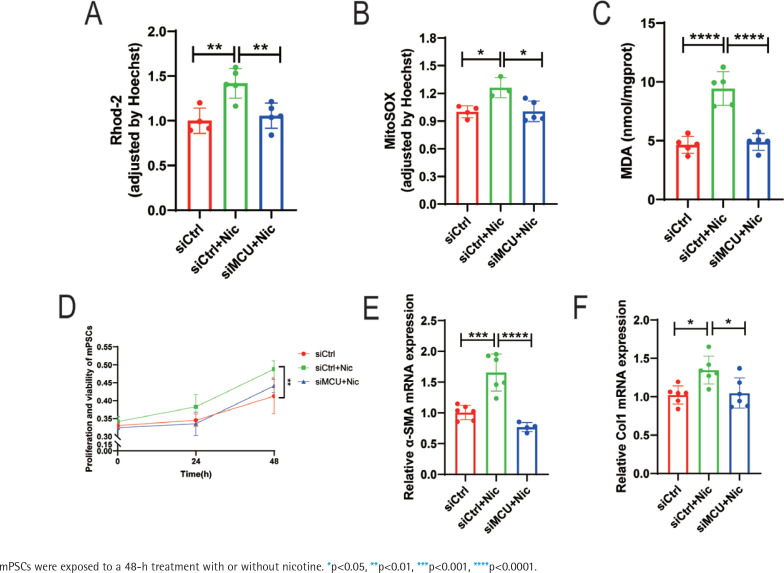
Nicotine aggravates mPSCs activation through the dysregulation of mitochondrial calcium homeostasis and oxidative stress mediated by MCU: A) Measurement of mitochondrial Ca^2+^ levels by normalizing the fluorescence intensity of Rhod-2 to that of Hoechst in mPSCs; B) Mitochondrial ROS levels in mPSCs; C) Intracellular MDA levels in mPSCs; D) Relative proliferation and viability of mPSCs determined by CCK-8 assays; E, F) mRNA levels of α-SMA and Col1 in mPSCs

Based on the preliminary findings, we employed siRNA transfection to knock down MCU in mPSCs to investigate its impact on nicotine-induced mPSCs activation and oxidative stress. The cells were divided into four groups: control group (siCtrl), nicotine group (siCtrl+Nic), MCU knockdown group (siMCU), and MCU knockdown + nicotine group (siMCU+Nic). Compared to the nicotine group, the application of nicotine after MCU knockdown resulted in a significant decrease in mitochondrial Ca^2+^ levels, whereas the sole knockdown of MCU did not exhibit significant changes in mitochondrial Ca^2+^ levels compared to the control group (p<0.01) (Figure 3A). Additionally, we investigated the impact of MCU on mitochondrial oxidative stress. MCU knockdown markedly attenuated the nicotine-induced elevation of mitochondrial ROS levels and intracellular MDA levels (p<0.05) (Figures 3B and 3C). The enhanced activation of mPSCs by nicotine was also suppressed by MCU knockdown. Cell proliferation, which was significantly increased after nicotine treatment (p<0.01), was reversed by MCU knockdown (Figure 3D). MCU knockdown effectively reversed the changes in mRNA levels of α-SMA and collagen I induced by nicotine (p<0.05) (Figures 3E and 3F) and alleviated the elevation of α-SMA protein levels (Supplementary file Figure S2A).

In addition, we investigated the effects of nicotine on mitochondrial dynamics-related proteins. Our results demonstrated that MCU knockdown significantly reduced the elevated mRNA levels of DRP1 in mPSCs stimulated by nicotine (p<0.0001) (Supplementary file Figure S2B). Furthermore, we utilized JC-1 to assess MMP and observed under a fluorescence microscope. JC-1 exhibited potential-dependent accumulation in mitochondria, and mitochondrial depolarization was indicated by a decrease in the red/green fluorescence intensity ratio in the aggregate form/monomer form. MCU knockdown alleviated the nicotine-induced reduction of MMP in mPSCs (Supplementary file Figure S2C).

Combining these findings with our previous research, we propose that nicotine may upregulate MCU, leading to increased mitochondrial calcium levels, exacerbation of mitochondrial oxidative stress, enhanced mitochondrial fission, and decreased MMP, ultimately promoting mPSCs activation.

### The MCU inhibitor Ru360 alleviates nicotine-aggravated pancreatic fibrosis in CP mice

Based on *in vitro* experiments, we further validated our findings in an *in vivo* experiment. We investigated the impact of nicotine on the severity of pancreatic fibrosis in CP mice and explored the therapeutic effects of the MCU inhibitor Ru360. We established a CP mouse model by intraperitoneal injection of cerulein and divided the CP mice into three groups: control group (Cae), nicotine group (Cae+Nic), and Ru360 group (Cae+Nic+Ru360) (Supplementary file Figure S3A). After sample collection, multiple parameters were analyzed, including body weight, pancreas weight, extent of pancreatic injury and fibrosis, activation of PSCs, and oxidative stress levels in pancreatic tissue.

**Figure 4:**
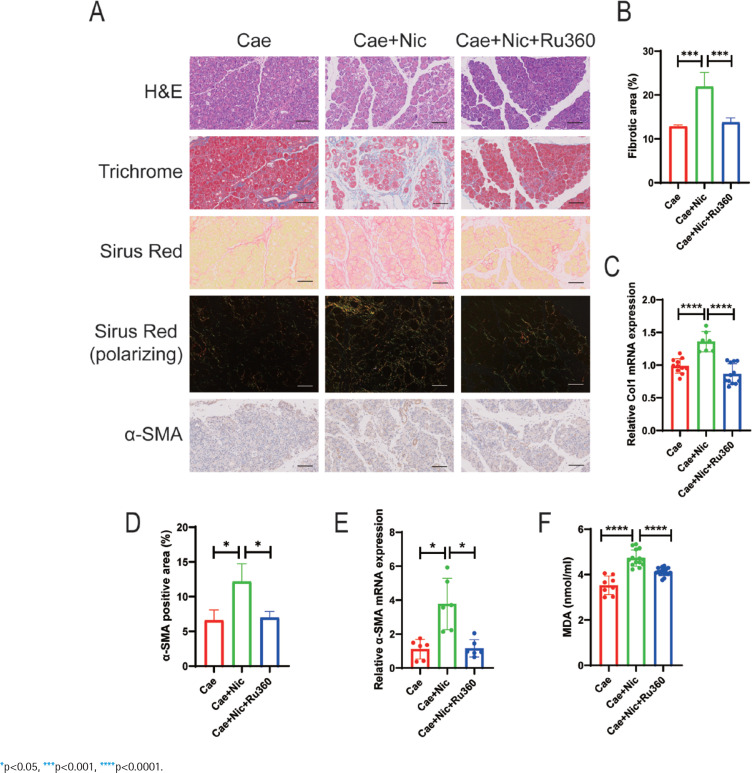
The MCU inhibitor Ru360 alleviates nicotine-aggravated pancreatic fibrosis in CP mice: A) Histological analysis of pancreatic tissues using H&E, Masson Trichrome, Sirus Red, and Sirus Red(polarizing) staining, along with immunohistochemical staining of α-SMA (scale bars=100 μm); B) Quantitative evaluation of the pancreatic fibrosis; C) mRNA levels of Col1 in pancreatic tissues; D) Immunohistochemical staining of α-SMA in pancreatic tissues; E) mRNA levels of α-SMA in pancreatic tissues; F) Measurement of serum MDA levels

The results demonstrated that nicotine significantly decreased the pancreas weight-to-body weight ratio in CP mice, which was effectively alleviated by the administration of Ru360 (p<0.05) (Supplementary file Figure S3B). Additionally, nicotine-induced histological changes in the pancreatic tissue, such as glandular atrophy and fibrosis, were observed (Figure 4A). Quantitative analysis of pancreatic fibrosis was conducted by measuring the positive area in Masson-trichrome staining, demonstrates that Ru360 significantly attenuated the exacerbated pancreatic fibrosis induced by nicotine (p<0.001) (Figure 4B). Furthermore, nicotine-induced upregulation of Col1 mRNA levels was significantly reversed by Ru360 (p<0.0001) (Figure 4C).

Moreover, Ru360 significantly inhibited the promoting effect of nicotine on PSCs activation. It effectively reversed the increased expression of α-SMA in pancreatic tissue induced by nicotine (p<0.05) (Figure 4D). This reversal was also evident at mRNA expression levels (p<0.05) (Figure 4E). Similarly, the serum levels of MDA were significantly elevated in the nicotine group, while the Ru360 group exhibited a significant reduction in MDA content (p<0.0001) (Figure 4F), indicating that Ru360 could reverse nicotine-aggravated oxidative stress damage in the pancreas of CP mice.

In summary, Ru360 effectively inhibits the nicotine-induced upregulation of MCU expression, thereby ameliorating oxidative stress damage in PSCs, ultimately alleviating the activation of PSCs and pancreatic fibrosis in CP mice exacerbated by nicotine.

## DISCUSSION

This study revealed that nicotine can upregulate the expression of MCU in mPSCs, leading to mitochondrial calcium overload and exacerbating oxidative stress, ultimately resulting in mPSCs activation. Conversely, knocking down MCU can reverse the aforementioned effects of nicotine. Similarly, nicotine can worsen pancreatic fibrosis and induce oxidative stress in the pancreatic tissue of CP mice, whereas treatment with the MCU inhibitor Ru360 can alleviate these conditions.

The harmful components of cigarette smoke exacerbate mitochondrial oxidative stress, which is believed to contribute to the aggravation of CP caused by smoking^23^. Our previous research has demonstrated that nicotine upregulates the expression of DRP1, a protein associated with mitochondrial fission mediated by mitochondrial oxidative stress, resulting in mitochondrial dynamic changes and dysfunction, ultimately promoting PSCs activation^7^. Similarly, studies have indicated that excessive production of ROS in PSCs can activate PSCs and stimulate collagen deposition by activating downstream AKT and NFκB pathways^24^. Animal experiments have also shown that the application of antioxidants can mitigate the activation and proliferation of rat PSCs induced by high sugar^25^. However, since ROS are produced during normal physiological activities, an increase in their levels alone may not accurately reflect oxidative stress damage. Therefore, this study further investigated the level of MDA, an index of oxidative stress damage.

Ca^2+^ overload is one of the key initiating factors for mitochondrial oxidative stress. Mutations associated with CP have been found to involve calcium-sensitive receptors, resulting in abnormal calcium signaling^26^. However, these mutations only increase susceptibility to CP, while the coexistence of toxic factors such as smoking, are the driving factors contributing to the onset and progression of CP^27^. Calcium signaling varies significantly between PSCs and pancreatic acinar cells. For instance, bradykinin can induce Ca^2+^ overload in PSCs while having no effect on Ca^2+^ concentration in neighboring pancreatic acinar cells^28^. As an important second messenger in signal transduction, calcium signaling has significant implications in various diseases. In nervous system diseases, mitochondrial calcium homeostasis plays a crucial role in maintaining the physiological function of neurons^29^. For instance, Alzheimer’s disease may be associated with dysregulation of mitochondrial calcium homeostasis^30,31^. Similarly, regulating calcium homeostasis has emerged as a novel target for malignant tumors^32^.

Mitochondrial calcium homeostasis is regulated by calcium transport-related proteins located on the mitochondrial inner membrane^33^. Numerous studies have investigated the mechanisms of calcium transport-related proteins, aiming to identify suitable therapeutic targets for diseases^18^. MCU is a multi-protein channel includes essential mitochondrial response element (EMRE), mitochondrial calcium uptake 1, 2, and 3 (MICU1/2/3)^34^. MICU1/2/3 are regulatory proteins that exert different modulatory effects on the channel^35^. This study revealed that knockdown of MCU alone did not significantly alter mitochondrial Ca^2+^ levels, but it resulted in reduced Ca^2+^ levels when Ca^2+^ levels were elevated. This suggests that the expression and function of MCU may be associated with intracellular and perimitochondrial Ca^2+^ levels.

### Limitations

This study has certain limitations. Firstly, the *in vivo* experiments did not involve the use of MCU gene knockout mice, and only one mitochondrial calcium-specific inhibitor was applied, which may limit the persuasiveness of the research findings. Additionally, we did not investigate the reciprocal interactions between intracellular Ca^2+^ and mitochondrial Ca^2+^. Therefore, further research is still needed to explore the underlying mechanisms of nicotine in CP progression.

## CONCLUSIONS

This study demonstrated that nicotine can modulate mitochondrial calcium homeostasis and oxidative stress by upregulating MCU expression, thereby promoting the activation of PSCs and excessive collagen production, leading to aggravated pancreatic fibrosis in CP. These findings may provide new insights into the pathogenesis of CP and represent potential therapeutic directions for the clinical management of nicotine-induced exacerbation of pancreatic fibrosis in CP.

## Supplementary Material



## Data Availability

The data supporting this research are available from the authors on reasonable request.
