# Supplementary material for: Nicotine aggravates pancreatic fibrosis in mice with chronic pancreatitis via mitochondrial calcium uniporter
Source: Tob Induc Dis. 2024 Apr 30;22:10.18332/tid/186587. doi: 10.18332/tid/186587 (PMC11057042; doi:10.18332/tid/186587)
Supplement: Supplementary file 1 [file TID-22-69-s1.pdf]

Supplementary material

Figure S1. The potential involvement of the calcium signaling pathway in the promotion of mPSCs activation by nicotine: A) Principal Component Analysis (PCA) reveals distinct differences between the nicotine group and the negative control group; B) Gene Set Enrichment Analysis (GSEA) demonstrates an increased enrichment of the calcium signaling pathway in the nicotine group compared to the negative control group (NES=1.272, *P*=0.046).

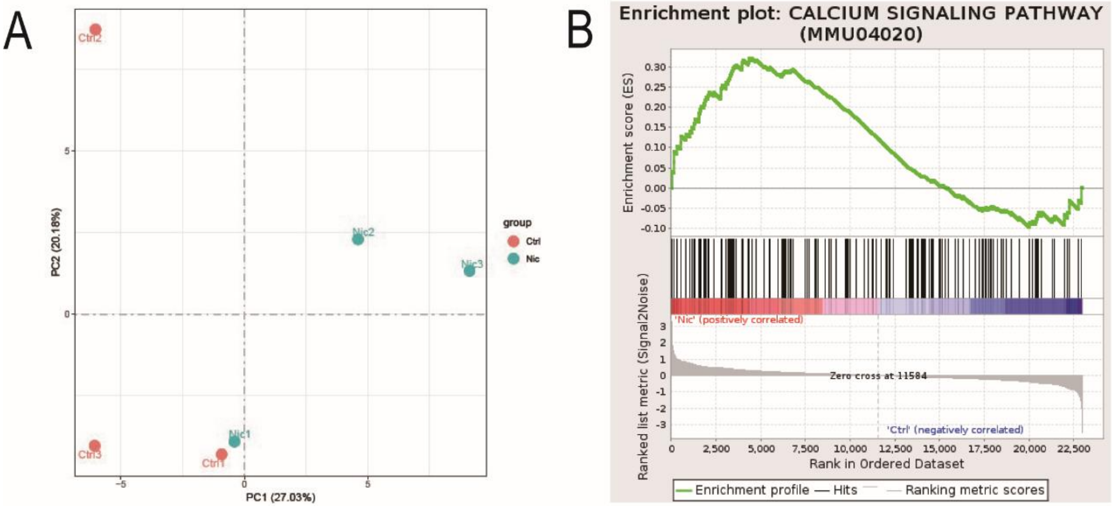

Figure S2. Nicotine aggravates mPSCs activation through the dysregulation of mitochondrial calcium homeostasis and oxidative stress mediated by MCU: A) Protein expression levels of MCU and α-SMA expression in mPSCs; B) mRNA levels of mitochondrial dynamin-related protein 1 (DRP1) in mPSCs; C) Mitochondria membrane potential (MMP) of mPSCs stained with JC-1 (fluorescent microscopy, scale bars=200μm). ****P<0.0001.

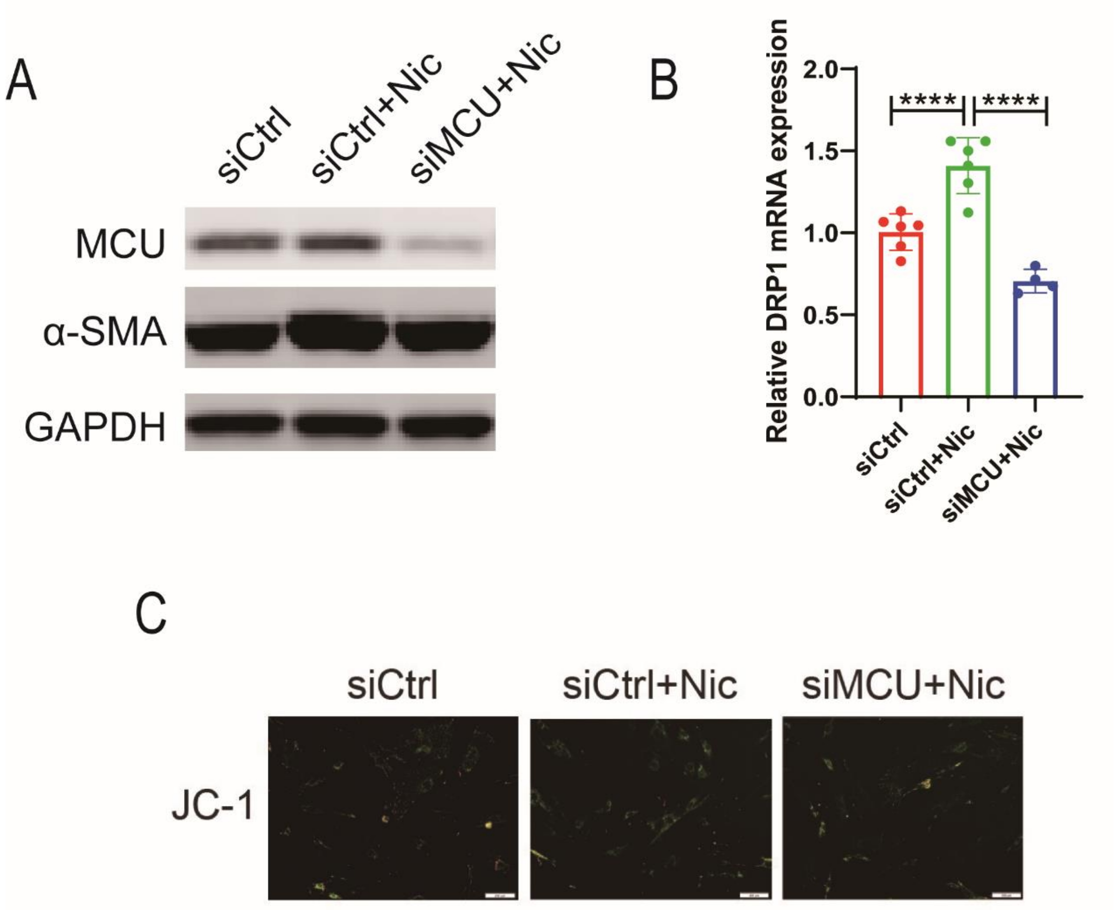

Figure S3. The MCU inhibitor Ru360 alleviates nicotine-aggravated pancreatic fibrosis in CP mice: A) Flow chart of the animal experiment (i.p., intraperitoneal injection; i.g., intragastric administration); B) Assessment of the pancreas-to-body weight ratio. **P*<0.05, ***P*<0.01.

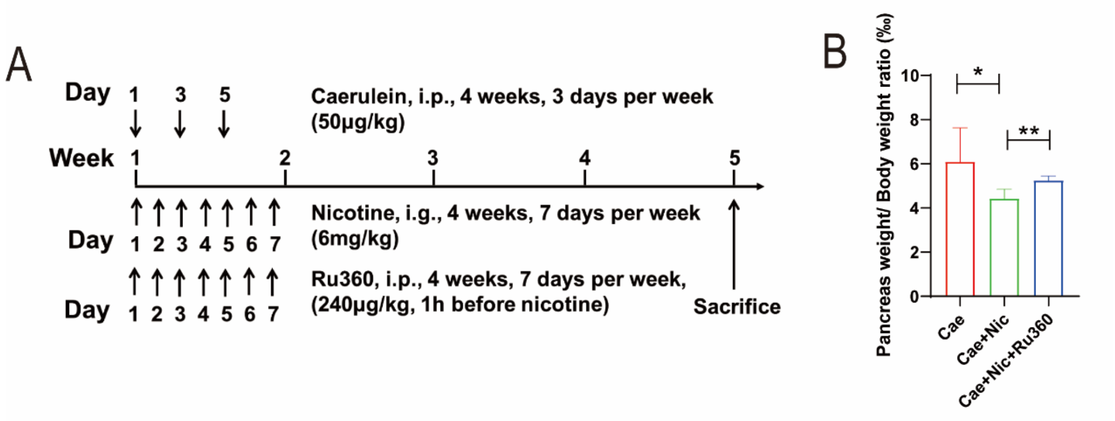

Supplementary Table 1. List of primers used for real-time PCR

Gene	Primer sequences (5' to 3')
HPRT	F: TCAGTCAACGGGGGACATAAA<br>R: GGGGCTGTACTGCTTAACCAG
β-actin	F: GTGACGTTGACATCCGTAAAGA<br>R: GCCGGACTCATCGTACTCC
α-SMA	F: TCCTGTTTCGGGAGCAGAAC<br>R: AGCTGGCCGTTCACTCTAAC
Col1	F: CGACCTCAAGATGTGCCACT<br>R: CCATCGGTCATGCTCTCTCC
MCU	F: GAGCCGCATATTGCAGTACG<br>R: CGAGAGGGTAGCCTCACAGAT
DRP1	F: TCAGATCGTCGTAGTGGGAA<br>R: TCTTCTGGTGAAACGTGGAC

F: Forward; R: Reverse.

Supplementary Table 2. List of antibodies for western blotting analysis.

Antibody	Catalog number	Species	Company
GAPDH	2118	Rabbit	Cell Signaling Technology
α-SMA	ab5694	Rabbit	Abcam
MCU	14997	Rabbit	Cell Signaling Technology
IRDye®: 680RD	926-68071	Goat anti-Rabbit	LI-COR

®: Trademark has been registered.

©2024 Wei X. et al.
